# Pan-cancer repository of validated natural and cryptic mRNA splicing mutations

**DOI:** 10.12688/f1000research.17204.3

**Published:** 2019-09-06

**Authors:** Ben C. Shirley, Eliseos J. Mucaki, Peter K. Rogan

**Affiliations:** 1 CytoGnomix Inc., London, Ontario, N5X 3X5, Canada; 2 Biochemistry, University of Western Ontario, London, Ontario, N6A 2C1, Canada; 3 Computer Science, University of Western Ontario, London, Ontario, N6A 2C1, Canada; 4 Oncology, University of Western Ontario, London, Ontario, N6A 2C1, Canada

**Keywords:** RNA Splice Sites, Single Nucleotide Polymorphism, Genome, Mutation, Chromosomes, Neoplasms, Information Theory, Next Generation Sequencing, Validation

## Abstract

We present a major public resource of mRNA splicing mutations validated according to multiple lines of evidence of abnormal gene expression. Likely mutations present in all tumor types reported in the Cancer Genome Atlas (TCGA) and the International Cancer Genome Consortium (ICGC) were identified based on the comparative strengths of splice sites in tumor versus normal genomes, and then validated by respectively comparing counts of splice junction spanning and abundance of transcript reads in RNA-Seq data from matched tissues and tumors lacking these mutations. The comprehensive resource features 341,486 of these validated mutations, the majority of which (69.9%) are not present in the Single Nucleotide Polymorphism Database (dbSNP 150). There are 131,347 unique mutations which weaken or abolish natural splice sites, and 222,071 mutations which strengthen cryptic splice sites (11,932 affect both simultaneously). 28,812 novel or rare flagged variants (with <1% population frequency in dbSNP) were observed in multiple tumor tissue types. An algorithm was developed to classify variants into splicing molecular phenotypes that integrates germline heterozygosity, degree of information change and impact on expression. The classification thresholds were calibrated against the ClinVar clinical database phenotypic assignments. Variants are partitioned into allele-specific alternative splicing, likely aberrant and aberrant splicing phenotypes. Single variants or chromosome ranges can be queried using a Global Alliance for Genomics and Health (GA4GH)-compliant, web-based Beacon “Validated Splicing Mutations” either separately or in aggregate alongside other Beacons through the public Beacon Network, as well as through our website. The website provides additional information, such as a visual representation of supporting RNAseq results, gene expression in the corresponding normal tissues, and splicing molecular phenotypes.

## Introduction

Next generation sequencing continues to reveal large numbers of novel variants whose impact cannot be interpreted from curated variant databases, or through reviews of peer-reviewed biomedical literature ^1^. This has created a largely unmet need for unequivocal sources of information regarding the molecular phenotypes and potential pathology of variants of unknown significance (VUS); in cancer genomes, such sources are critically needed to assist in distinguishing driver mutations from overwhelming numbers of bystander mutations. VUS classification criteria highlight the limitations in genome interpretation due to ambiguous variant interpretation. Of the 458,899 variant submissions in NCBI’s ClinVar database with clinical interpretations, nearly half (n=221,271) are VUS (as of November 5th 2018). Only 10,784 variants in ClinVar have been documented to affect mRNA splicing at splice donor or acceptor sites, with 1,063 of these being classified as VUS, and cryptic mRNA splicing mutations are not explicitly described. The current ACMG criteria ^2^ for variant pathogenicity prevent clinical classification of most VUS. Functional evidence that VUS either disrupt or abolish expression of genes has been sought to improve classification and provide insight into the roles, if any, of individual VUS in predisposing or causing disease. We present a comprehensive data repository for a relatively common mutation type (cis-acting variants that alter mRNA splicing). Mutations are predicted with information theory-based analyses ^3^, and supported with functional evidence that variants in tumor genomes are specifically associated with abnormally spliced mRNAs that are infrequent or absent in transciptomes lacking these variants ^4^.

Information theory (IT) has been proven to accurately predict impact of mutations on mRNA splicing, and has been used to interpret coding and non-coding mutations that alter mRNA splicing in both common and rare diseases ^3, 5– 15^. We have described an IT-based framework for the interpretation and prioritization of non-coding variants of uncertain significance, which has been validated in multiple studies involving novel variants in patients with history or predisposition to heritable breast and/or ovarian cancer ^11– 15^.

The Cancer Genome Atlas (TCGA) Pan-Cancer Atlas (PCA) is a comprehensive integrated genomic and transcriptomic resource containing data from >10,000 tumors across 33 different tumor types ^16^. Here, we utilized IT-based tools for assessment of high quality sequenced variants in TCGA patients, as well as patients from tumor datasets provided by the International Cancer Genome Consortium (chronic lymphocytic leukemia, esophageal adenocarcinoma, malignant lymphoma, pancreatic cancer endocrine neoplasms, as well as liver, ovarian, and renal cell cancers), for their potential impact on mRNA splicing. The accuracy of predicted mutations was evaluated with the algorithm we previously developed ^4^ that compares transcripts from cases carrying these variants with others lacking them. The results of these genome-wide analyses are presented using an online internet resource, ValidSpliceMut, which can also be queried through the Beacon Network ^17, 18^.

## Methods

### TCGA and ICGC data acquisition and processing

Controlled-access data was obtained with permission from the Data Access Committee at NIH for TCGA and from the International Cancer Genomics Consortium. Patient RNA sequencing BAM files (tumor and normal, when available) and their associated VCF files (GRCh37) were initially obtained from the CancerGenomeHub (CGhub). Files were later downloaded through Genomic Data Commons using the GDC Data Transfer Tool (version 1.3.0), as CGhub was decommissioned mid-project. Genomic data from ICGC was downloaded through the Score client (version 1.5.0). Variants in VCF files which did not pass quality control (QC) were not analyzed.

### Information analysis and RNA-Seq validation of splicing variants

We used the *Shannon Pipeline* software (SP; which applies IT to rapidly perform high-throughput, *in silico* prediction of the impacts of variants on mRNA splicing) ^19^ to analyze all QC-passing variants in VCFs from TCGA and ICGC (>168 million TCGA and >41 million ICGC variants) to evaluate their potential impact on splice site binding strength (changes in information content, *R _i_*). *R _i_* is a measure of binding site strength; it is related to affinity through the second law of thermodynamics and is measured in bits (a glossary of IT and Veridical terms can be found here). According to Shannon information theory, only sites with *R _i_* values exceeding zero bits can be bound. The minimum fold change in affinity is exponentially related to the difference in *R _i_* values of wild type and mutant binding sites (≥ 2 ^Δ *Ri*^). For example, a 3 bit change would result in at least an 8-fold change in binding affinity. Variants which were predicted to strengthen known natural sites or weaken cryptic splice sites were excluded from all subsequent analyses. We also required novel cryptic splice sites to be strengthened by ≥ 2 bits (at least 4 fold), and with final strengths ( *R _i,final_*) exceeding that of the nearest natural site of the same polarity.

To validate the potential impact of SP-flagged mutations, *Veridical* software analyzed genomic variants (including insertions and deletions) by comparing the RNA-Seq alignment in the region surrounding the variant in the index case with the corresponding interval in control transcriptomes (normal and tumor tissue of the same type) lacking the same variant ^4, 20^. The Veridical algorithm: a) counts abnormally spliced reads in RNA-Seq data (categorized as either cryptic site use, exon skipping, or intron inclusion [containing or adjacent to the flagged mutation]), b) applies the Yeo-Johnson transformation to these results, and c) determines the null hypothesis probability (p-value) that the transformed read count corresponds to normal splicing. In tumor types where normal controls were not available, a set of RNA-Seq datasets from 100 different normal tissues from TCGA were used (e.g. a combination of 5 tissue types: BRCA, BLCA, LUAD, KIRC, PRAD). Variants not flagged for any particular evidence type (p-value > 0.05) were inconsistent with being splicing mutations, and were considered alternatively spliced and most likely, benign. After this analysis, Veridical validated 341,486 unique mutations for their direct impact on mRNA splicing ( Table 1). The Shannon pipeline-flagged and Veridical-filtered results were combined into a single large table ( *Dataset 1* ^21^) that contains the source data for the ValidSpliceMut SQL database and the associated Beacon application.

**Table 1.** Unique Flagged Variants by TCGA and ICGC Tumor Tissue Type *.

**TCGA-ACC**	**TCGA-BLCA**	**TCGA-BRCA**	**TCGA-CESC**	**TCGA-CHOL**	**TCGA-COAD**	**TCGA-DLBC**
1,717	9,865	24,181	25,822	9,817	7,512	6,036
**TCGA-ESCA**	**TCGA-GBM**	**TCGA-HNSC**	**TCGA-KICH**	**TCGA-KIRC**	**TCGA-KIRP**	**TCGA-LAML**
19,361	935	2,840	26,519	6,711	4,892	19,503
**TCGA-LGG**	**TCGA-LIHC**	**TCGA-LUAD**	**TCGA-LUSC**	**TCGA-MESO**	**TCGA-OV**	**TCGA-PAAD**
1,346	12,461	18,262	2,628	303	88,136	1,585
**TCGA-PCPG**	**TCGA-PRAD**	**TCGA-READ**	**TCGA-SARC**	**TCGA-SKCM**	**TCGA-STAD**	**TCGA-TGCT**
90	944	3,083	20,024	12,515	20,245	467
**TCGA-THCA**	**TCGA-THYM**	**TCGA-UCEC**	**TCGA-UCS**	**TCGA-UVM**	**ICGC-CLLE**	**ICGC-ESAD**
56,962	16,599	28,524	10,716	2,498	2,041	61
**ICGC-LIRI**	**ICGC-MALY**	**ICGC-OV**	**ICGC-PACA**	**ICGC-RECA**		
2,255	2,652	2,818	3,182	4,255		

*The number of Veridical-flagged mutations in each TCGA and ICGC cancer data sets. Variants shared between multiple tissue types were counted for each category. Variant and RNA-Seq data were provided by The Cancer Genome Atlas Pan-Cancer Analysis Project ^16^. **TCGA:** ACC [Adrenocortical carcinoma], BLCA [Bladder Urothelial], BRCA [Breast Cancer], CESC [Cervical Squamous Cell Carcinoma], CHOL [Cholangiocarcinoma], COAD [Colon Adenocarcinoma], DLBC [Lymphoid Neoplasm Diffuse Large B-cell Lymphoma], ESCA [Esophageal Cancer], GBM [Brain Glioblastoma Multiforme], HNSC [Head and Neck Squamous Cell Carcinoma], KICH [Kidney Chromophobe], KIRC [Kidney Renal Clear Cell Carcinoma], KIRP [Kidney Renal Papillary Cell Carcinoma], LAML [Acute Myeloid Leukemia], LGG [Brain Lower Grade Glioma], LIHC [Liver Hepatocellular carcinoma], LUAD [Lung Adenocarcinoma], LUSC [Lung Squamous Cell Carcinoma], MESO [Mesothelioma], OV [Ovarian Serous Cystadenocarcinoma], PAAD [Pancreatic Cancer], PCPG [Pheochromocytoma and Paraganglioma], PRAD [Prostate Adenocarcinoma], READ [Rectum Adenocarcinoma], SARC [Sarcoma], SKCM [Skin Cutaneous melanoma], STAD [Gastric Adenocarcinoma], TGCT [Testicular Germ Cell Tumors], THCA [Head and Neck Thyroid Carcinoma], THYM [Thymoma], UCEC [Uterine Corpus Endometrial Carcinoma], UCS [Uterine Carcinosarcoma], UVM [Uveal Melanoma]. **ICGC:** CLLE [Chronic Lymphocytic Leukemia], ESAD [Esophageal Adenocarcinoma], LIRI [Liver Cancer], MALY [Malignant Lymphoma], OV [Ovarian Cancer], PACA [Pancreatic Cancer Endocrine Neoplasms], RECA [Renal Cell Cancer].

### Development of the ValidSpliceMut database and Beacon

We created a publicly accessible Application Programming Interface (API) ( https://beacon.cytognomix.com) that can be utilized to programmatically query variants passing filter thresholds described above ( *Dataset 1* ^21^). It was built in accordance with the GA4GH Beacon v1.0.0 specification, which describes a Representational State Transfer (REST) API for genetic data sharing ^22^. A Beacon accepts queries using an HTTP request and returns JavaScript Object Notation (JSON). Our Beacon implementation is coded in PHP 7.0 and utilizes a MySQL database (version: 5.7.24) with indexes applied to variant ID, chromosome, and coordinate fields (GRCh37). The returned JSON object reports whether the variant was found within our Beacon dataset as well as metadata including splice site coordinate, splice type, site type, the IT-based measures *R _i,initial_* and *R _i,final_*, affected individual IDs, tumor type, Veridical evidence by type annotated with significance level, and, if known, the corresponding rsID with its average heterozygosity (dbSNP 150). The metadata for each variant sent to the Beacon Network is a concise subset of available results in our database. It includes the first relevant database entry, meaning that if the variant exists within multiple cases only the first will contribute fields to the metadata. However, among this metadata is a hyperlink to our local website containing results for any remaining tumors.

**Figure 1.**
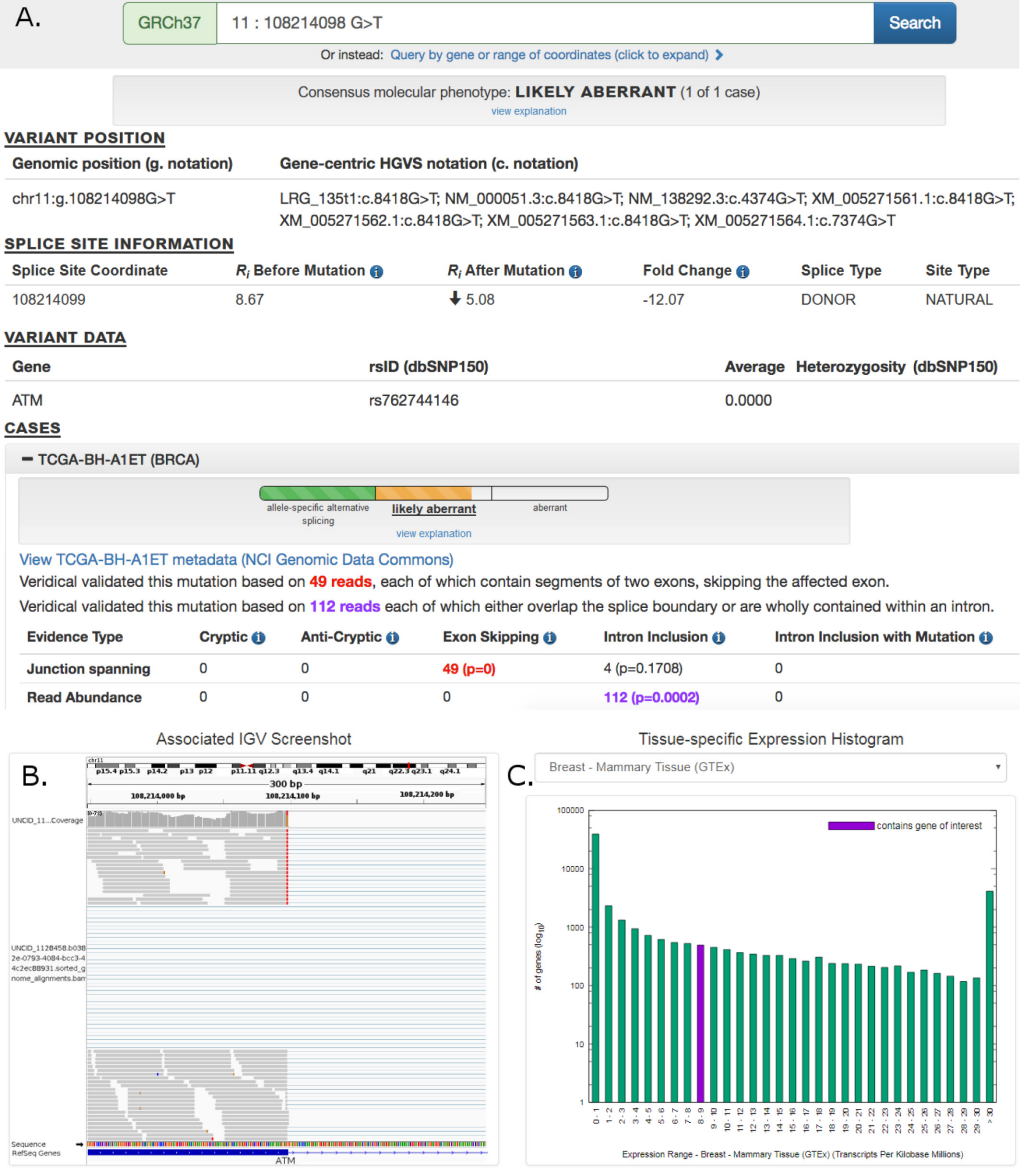
Screenshot of *ATM*:g.108214098G>T Results Provided By ValidSpliceMut Website. ( **A**) At the top of the page, the predicted molecular phenotype of the mutation for all cases is presented (prediction algorithm is shown in Figure 2). Then, the ‘Variant Position’ heading displays the variant of interest in g. notation, and provides a link which queries the Mutalyzer API to obtain the variant coordinate in a gene-centric c. mutation format. Variant-specific and splice site-specific tabular results are presented under the headings “Splice Site Information” and “Variant Data”. Results are then organized by TCGA and ICGC sample IDs (‘cases’) harboring the mutation within a series of expandable panels. A link is provided to patient tumor metadata on the GDC data portal. Each panel consists of the molecular phenotype classification for that particular sample, and the read counts and p-values for each Veridical evidence type. Significant p-values (≤ 0.05) are highlighted in bold. Evidence types deemed “strongly corroborating” (Viner *et al*. 2014) are color coded and correspond to the dynamically generated text appearing above the table. ( **B**) An integrative genome viewer (IGV) image showing alignment of expressed sequence reads. IGV screenshots are provided only for mutations present <1% of population (in dbSNP 150), with ≥ 5 junction-spanning reads, and are highly significant (p < 0.01) for cryptic splicing, exon skipping, and/or intron inclusion with mutation. A specific IGV screenshot for this sample captures the region surrounding the mutation. Here, several RNA-Seq reads show skipping of the affected exon. ( **C**) A dynamically generated histogram presents expression levels of all genes for a selected normal tissue type. Genes are grouped into bins based on expression level, denoted on the x-axis. The number of genes present in each bin is shown on the y-axis (log _10_ scale). The histogram key indicates the expression range which contains the variant-containing gene (purple). Tissue type can be changed via a drop-down list.

We developed the website, ValidSpliceMut (example output indicated in Figure 1) to serve as a local interface to our Beacon, allowing users to manually search for a variant, by gene name or genome coordinate range. ValidSpliceMut automatically queries our Beacon, and formats the results of the search, if any. This website provides a complete view of variants, including Veridical-based evidence on all data related to every affected individual. If a variant is associated with multiple splice sites, the user is presented with a brief overview of all affected sites and must select a desired site to continue. To obtain the coordinate of the queried variant in gene-centric notation, a link is provided which queries the Mutalyzer API and generates coordinates for all available transcripts. ValidSpliceMut only reports transcripts for the gene affected by the variant.

A results page presents variant-specific data in tabular format and an expandable list of panels describing the affected cases. Each of these panels contains Veridical output in tabular format for the selected tumor, a link to the tumor metadata at US National Cancer Institute (by querying the GDC API to obtain a UUID which is used to construct a link to the GDC data portal), the predicted molecular phenotype for that case, an Integrative Genome Viewer (IGV) screenshot containing the variant (IGV screenshots are available for selected variants, see below), and a histogram which presents the expression levels of the variant-containing gene compared to all other gene expression levels across a selected normal tissue type (created dynamically using gnuplot 5.0). The tissue expression data is provided by GTEx (downloaded on 10/22/18). However, several tumor types did not have a GTEx equivalent (TCGA: CHOL, DLBC, MESO, READ, SARC, THYM and UVM; ICGC: MALY). The GNF Expression Atlas 2 ^23^ was downloaded from the UCSC Genome Browser and was used for expression data for both lymph nodes (DLBC; MALY) and the thymus (THYM). For the remaining tissues, expression data from the following studies were obtained from the Genome Expression Omnibus (GEO): GSE76297 (CHOL), GSE2549 (MESO), GSE15781 (READ) GSE44426 (SARC), and GSE44295 (UVM).

**Figure 2.**
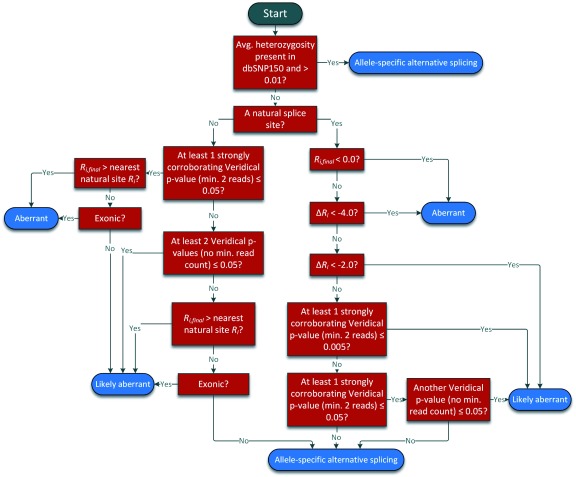
Evidence-Based Case Classification Flowchart. Flowchart depicting steps taken to classify the molecular phenotype of each case. Cryptic site (left) and natural site changes (right) have differing categorization criteria, which involve the combination of information theory-based predictions and Veridical evidence. SNPs common in dbSNP150 (>0.01 average heterozygosity) are immediately considered allele-specific alternative splicing.

ValidSpliceMut quantifies information analyses, expression pattern evidence and allele frequencies for each variant. We derived an algorithm to classify the consequences of each case, i.e. a tumour with a particular mutation, based on these properties. The molecular phenotypes assigned can be either (1) aberrantly spliced, (2) likely aberrantly spliced, or (3) result in allele-specific alternative splicing. The thresholds and parameters of this classification system ( Figure 2) were first calibrated against the clinically-validated phenotypes of variants in the ClinVar database. ClinVar contains 34,671 variants that are also present in ValidSpliceMut. In ClinVar, 1,948 of these variants are designated as pathogenic (1,180 natural; 768 cryptic) and 26,056 variants as benign (affecting 9,601 natural and 16,455 cryptic splice sites). We determined if discernible characteristics of these splicing mutations in ValidSpliceMut corresponded to either pathogenic or benign designations in ClinVar. Pathogenic variants in ClinVar were considered to be equivalent to aberrant splice molecular phenotypes in ValidSpliceMut. Benign variants in ClinVar were considered to be equivalent to allele-specific alternative splicing in ValidSpliceMut. From our previous work on information-theory based analysis of splicing mutations ^3^, the characteristics evaluated for splice sites associated or altered by each variant included: 1) natural site Δ *R _i_* value, 2) cryptic site Δ *R _i_*, 3) cryptic site *R _i_* strength relative to the *R _i_* value of the nearest cognate natural site, 4) the number of supporting evidence types detected by Veridical, as well as their respective p-values, 5) the heterozygosity of variant in dbSNP150, if present in this database, 6) the overall level of expression in the tissue type from which the tumor was derived, and 7) the distance of the cryptic site, if present, from the closest cognate natural splice site. All of the characteristics were assigned a weight between 0 and 1 for each variant; natural and cryptic splicing mutations were examined separately. For characteristics relevant to a particular site type (4 of these characteristics were relevant for natural splice sites, 6 were for cryptic sites), all potential combinations of weights divisible by 0.2 were examined. All characteristics were initially classified as either aberrantly or allele-specific alternatively spliced; subsequently, the weighted majority of classifications were computed for each variant in each tumour. The Matthews correlation coefficient (MCC) was chosen to measure adherence to ClinVar classifications for each weighting scheme as it can account for the imbalance in ClinVar between number of variants classified as benign versus those indicated as pathogenic in assessing the performance of each characteristic. Examination of natural splice site variants alone demonstrated that characteristics 1 and 5 together were consistently the best predictors of ClinVar phenotypes (MCC: 0.777) with Δ *R _i_* < -2.7 bits [aberrant characteristic] and average heterozygosity ≤ 0.002 [aberrant characteristic]) as the optimal thresholds. Other variant characteristics, either individually or in combination with characteristics 1 and 5, had significantly lower concordance with ClinVar designations (for example, inclusion of tissue specific expression levels reduced MCC to 0.424, and inclusion of aberrant expression patterns based on Veridical results decreased this to 0.446).

Variants known to be frequent in dbSNP150 (>0.01 average heterozygosity) are initially partitioned by the algorithm into allele-specific alternative splicing phenotype, since they are indistinguishable from germline polymorphisms. Indeed, the ClinVar database indicates that such variants also present in ValidSpliceMut were 182-fold more likely to be benign than pathogenic, in contrast with 3-fold for mutations with <0.01 average heterozygosity. Since variants with benign phenotypes may also be infrequent (heterozygosity ≤ 0.01), a threshold Δ *R _i_* value that optimally distinguished benign from pathogenic natural splice site variants was determined among those also present in ValidSpliceMut. Variants that decrease natural splice site strength (Δ *R _i_*) by ≥ 4 bits (or ≥16 fold) nearly completely exclude all benign variants in ClinVar (< 4.7%). The threshold is robust, as the percentage of benign variants steeply decreases from Δ *R _i_* < -1 (44.9%) to Δ *R _i_* < -4 bits (4.7%), then level out with larger reductions in Δ *R _i_*. This criterion prevented many benign variants from being classified as causing either aberrant or likely aberrant molecular phenotypes ( Figure 2).

Inferred molecular phenotypes in ValidSpliceMut are corroborated by previously published information theory-based analyses. Natural splice sites containing variants with modestly decreased *R _i_* values (of ≤ 2 bits) do not detectably alter mRNA splicing ^24^. Such small changes in *R _i_* values have been consistently associated with benign genetic polymorphisms ^8, 9^. Conversely, splicing mutations with Δ *R _i_* < -7 bits result in severe phenotypes in inherited disorders ^25, 26^. Activated cryptic splice sites that result in novel mRNA isoforms exhibit *R _i_* values competitive with adjacent natural splice sites of the same polarity ^6, 9^. Exonic cryptic splice sites are more likely to be classified as either aberrant or likely aberrant than intronic cryptic sites. Exon recognition by spliceosomes is directional and processive from the natural acceptor splice site to the first downstream donor site with suitable strength to form an exon, and would therefore favor exonic cryptic site use ^9^. Acceptor cryptic sites that overlap with these cognate natural sites can also impair exon recognition ^4, 20^, inducing exon skipping ^7, 27^ and/or intron inclusion ^4, 20^.

After partitioning variants based on heterozygosity and Δ *R _i_* values, the algorithm applies supporting Veridical evidence types to molecular phenotype assignments. Evidence for aberrant splicing deemed "strongly corroborating" (for example, junction read types) is weighted more heavily than other evidence types ^4^, thus, an "aberrant" or "likely aberrant" classification is more likely in the presence of a strongly corroborating evidence type with p -value ≤ 0.05. Multiple Veridical evidence types (at least one of which is required to be strongly corroborating) with p-values ≤ 0.05, or the presence of a strongly corroborating evidence type with ≤ 0.005, reinforces a Veridical prediction of aberrant splicing.

When displaying the predicted molecular phenotype classification for each case, the classifications of other splice sites affected by the same variant in the same tumour type are also examined. If any of these other sites cause a case to be classified as aberrant, all sites affected by this variant are uniformly classified as aberrant. Reclassification affected a minor fraction of variants in ValidSpliceMut. Slightly over 10-fold more of the reclassified variants were upgraded from likely aberrant to aberrant compared to variants reclassified allele-specific to aberrant, where n=30,733 [11% of all aberrant cases] were reclassified as aberrant from likely aberrant, while n=3,026 [1% of aberrant cases] were reclassified from allele-specific alternative splicing. The majority of reclassified mutations affect cryptic sites, relative to natural site-altering mutations (11.3% and 0.7% of all aberrant cases, respectively). In the event reclassification has occurred, this is denoted below the classification evidence bar associated with the case.

Although individual cases are assigned according to their distinct molecular phenotypes, there are many combinations of evidence types which can lead to the same phenotypic assignment. During classification, all evidence types are calculated first, then the algorithm applies a series of rules are applied to arrive at an unambiguous classification of each case. This is depicted with a contiguous evidence bar (shown in Figure 1), which linearly arranges the classifications of each variant according to phenotypic severity of the effect on mRNA splicing. The evidence bar is constructed using a point-based system which considers all sources of evidence pertaining to the type of splice site affected by the variant. The position of the bar within a phenotypic classification reflects the balance between evidence supporting and contrasting the assigned classification. Supporting evidence moves the bar to the right, while contrasting evidence moves the bar to the left within a classification category. Equal levels of supporting and contrasting evidence types will cause the evidence bar to appear in the middle of the classification category.

After classifying all cases containing the specified variant (regardless of tumour type), the most frequent classification among all cases, termed the consensus molecular phenotype, is summarized and displayed at the top of the Results page (example in Figure 1). Adjacent to the consensus molecular phenotype, the number of cases assigned the most common classification is indicated alongside the total number of cases. Multiple consensus phenotypes are presented if there is an identical number of cases in each of the classification categories.

To generate IGV images presented on the webpage, a bash script was written to automatically load the RNA-Seq BAM file of a patient with a mutation of interest into IGV, set the viewing window within the region of interest (300nt window, centered on the variant), sorted to bring reads containing the variant of interest to the top of the screen (to increase chance of visualizing mutant splice form), followed by a screen capture. The generation and storage of IGV images for all patient-mutation pairs would be prohibitive due to limitations in time and server space requirements. Therefore IGV images showing evidence of splicing abnormalities were generated *only* for patient-mutation pairs which met the most stringent criteria: the mutation was required to be flagged for junction-spanning cryptic site use, exon skipping, or intron inclusion (with mutation); the flagged category must include 5 or more reads in this category; if the variant is present in the dbSNP database (release 150), the frequency was required to be < 1% of the population; and the Veridical results, in which the mutations flagged were required to exhibit p ≤ 0.01 for at least one form of evidence of a splicing abnormality. In some cases, the splicing event observed by Veridical may not be present (or displayed in its entirety) within the image window as the automated procedure used to create these images does not present all evidential sequence reads due to limitations on the number of reads that can be shown. Additionally, reads appearing as exon skipping may instead indicate a pre-existing cryptic site outside of the viewing window (for examples, see Table 2; *FAT1*:g.187521515C>A [c.11641-1G>T] and *SMAD3*:g.67482748C>G [c.1155-3C>G]).

**Table 2.** Representative Validated Splicing Mutations in COSMIC Cancer Gene Census genes.

Gene	Splice Mutation	*R _i_*(bits)	Tumor	Observed Splicing Event
*CASC5*	15:40942786G>A (c.6212+5G>A)	4.8 > 1.7 (Natural Site)	AML	The natural donor site of *CASC5* exon 19 (NM_144508.4) is weakened, leading to a significant increase in intron inclusion.
*DNMT3A*	2:25467022A>G (c.1851+2T>C)	3.6 > -3.5 (Natural Site)	AML	The natural donor site of *DNMT3A* exon 15 (NM_022552.4) is abolished, resulting in a significant increase in total exon skipping and intron inclusion.
*STAG2*	X:123176495G>A (c.462G>A)	6.5 > 3.5 (Natural Site)	BLCA	The natural donor of *STAG2* exon 6 (NM_006603.4) is weakened, and a significant amount of exon 6 skipping is observed.
*STAG2*	X:123200024G>A (c.2097-1G>A)	19.5 > 8.6 (Natural Site)	BLCA	The natural acceptor of *STAG2* exon 21 (NM_006603.4) is weakened, resulting in a significant increase in exon 21 skipping.
*ATM*	11:108214098G>T (c.8418G>T)	8.7 > 5.1 (Natural Site)	BRCA	A natural donor site is weakened, leading to a significant increase in *ATM* exon 57 (NM_000051.3) skipping events. Some reads with mutation depict wildtype, leaky splicing.
*BARD1*	2:215645882A>T (c.716T>A)	0.9 > 3.1 (Cryptic Site)	BRCA	The mutation strengthens a cryptic site within *BARD1* exon 4 (NM_000465.2). Reads which use this activated cryptic site contain the mutation (one exception). Some reads with mutation depict wildtype, leaky splicing.
*GATA3*	10:8115701G>C (c.1048-1G>C)	0.9 > -10.7 (Natural Site)	BRCA	The mutation abolishes the natural acceptor of *GATA3* exon 6 (NM_002051.2). This both increases the use of a pre-existing exonic cryptic splice site (4.2 > 5.6 bits; leads to an 8nt deletion) and significantly increases overall intron inclusion.
*TP53*	17:7577609C>T (c.673-1G>A)	6.0 > -4.9 (Natural Site)	BRCA	A natural acceptor site is abolished, activating a cryptic site 49nt upstream ( *R _i_*=5.2 bits) of *TP53* exon 7 ** (NM_000546.5).
*POLD1*	19:50920353A>G (c.3119A>G)	8.6 > 6.1 (Natural Site)	COAD	The natural donor of *POLD1* exon 25 (NM_002691.3) is weakened, leading to a significant increase in overall exon skipping.
*SMAD3*	15:67482748C>G (c.1155-3C>G)	11.9 > 3.1|-4.0 > 7.7 ( Natural | Cryptic)	COAD	This mutation weakens the natural acceptor of *SMAD3* exon 9 (NM_005902.3) and predicts a cryptic site that does not appear to be used. A significant number of intron inclusion reads are observed. A distant pre-existing cryptic acceptor (9.6 bits; 3,598nt from natural acceptor) was used in multiple reads.
*PIK3R1*	5:67591246A>G (c.936-2A>G)	7.5 > -7.3 (Natural Site)	GBM	The natural acceptor of *PIK3R1* exon 8 (NM_181504.3) is abolished, which promotes a significant increase in exon 8 skipping.
*FAT1*	4:187521515C>A (c.11641-1G>T)	5.3 > -2.4 (Natural Site)	HNSC	The natural acceptor of *FAT1* exon 22 (NM_005245.3) is abolished, resulting in both intron inclusion (overall intron inclusion and the use of a 2.3 bit cryptic site 82nt upstream of natural acceptor) and use of two exonic cryptic sites (237nt and 234nt from the natural acceptor; *R _i_*=1.0 bits and -0.2 bits, respectively).
*TGFBR2*	3:30729875G>A (c.1397-1G>A)	8.4 > -2.5 (Natural Site)	HNSC	*TGFBR2* exon 6 natural acceptor (NM_003242.5) is abolished, leading to multiple splicing events: intron inclusion, use of three cryptic sites (35nt exonic [ *R _i_*=3.7 bits], 30nt and 972nt intronic [ *R _i_*=0.4 bits and 11.2 bits, respectively]), and exon 6 and 7 skipping (activates a novel pseudo exon ^~^55kb downstream of exon 7).
*PBRM1*	3:52682355C>G (c.813+5G>C)	6.8 > 2.9 (Natural Site)	KIRC	The natural donor of *PBRM1* exon 8 (NM_018313.4) is weakened, which leads to a significant increase in exon 8 skipping.
*PBRM1*	3:52685756A>G (c.714+2T>C)	7.7 > 0.7 (Natural Site)	KIRC	The natural donor of *PBRM1* exon 7 (NM_018313.4) is abolished, resulting in a significant increase in exon skipping.
*SETD2*	3:47079269T>A (c.7239-2A>T)	9.8 > 2.1| 6.4 > 9.0 ( Natural | Cryptic)	KIRC	This mutation both significantly weakens the natural acceptor of *SETD2* exon 18 (NM_014159.6) while strengthening a 4nt exonic cryptic site, which is used.
*RB1*	13:49027249T>A (c.1814+2T>A)	4.9 >-13.7 (Natural Site)	LUAD	The natural donor of *RB1* exon 18 (NM_000321.2) is abolished, leading to a significant increase in both exon skipping and intron inclusion. All intron inclusion reads contain the mutation of interest.
*RBM10*	X:47006900G>T (c.17+3G>T)	7.8 > 4.1 (Natural Site)	LUAD	The natural donor of *RBM10* exon 2 (NM_005676.4) is weakened, leading to a significant increase in exon 2 skipping.
*RBM10*	X:47028898G>T (c.201+1G>T)	8.7 > -9.9 (Natural Site)	LUAD	*RBM10* exon 3 (NM_005676.4) natural donor is abolished. Reads which overlap the exon-intron junction are observed (all reads contain mutation). Use of cryptic donor (61nt upstream of donor; *R _i_*=1.7 bits) is observed as well.
*DDX5*	17:62500098 TACAG>T (c.441+2delACAG)	-1.3 > 5.4 (Cryptic Site)	PRAD	The mutation creates a 5.4 bit cryptic donor within *DDX5* exon 4 (NM_004396.3), which would lead to a 4nt deletion of exon 4. Note that wildtype splicing is still the dominant isoform observed.
*PTEN*	10:89690802G>A (c.210-1G>A)	8.5 > -2.3 (Natural Site)	PRAD	The natural acceptor of *PTEN* exon 5 (NM_000314.4) is abolished, leading to an increased amount of exon 5 skipping.
*NRAS*	1:115258669A>G (c.111+2T>C)	8.1 > 1.1 (Natural Site)	SKCM	The mutation abolishes the natural donor of *NRAS* exon 2 (NM_002524.4), which promotes a significant increase in exon 2 skipping.
*PPP6C*	9:127933364C>T (c.171G>A)	6.7 > 3.7 (Natural Site)	SKCM	The mutation weakens *PPP6C* exon 2 (NM_002721.4) natural donor, leading to increased intron inclusion. All reads which cross the splice junction contain the mutation. An intronic cryptic site is also activated (110nt downstream; *R _i_*=6.4 bits).
*PPP6C*	9:127923119C>G (c.237+1G>C)	6.8 > -11.8 (Natural Site)	SKCM	This mutation abolishes the natural donor of *PPP6C* exon 3 (NM_002721.4), resulting in a significant increase in exon 3 skipping.
*BAP1*	3:52442512T>C (c.233A>G)	1.9 > 5.1 (Cryptic Site)	UVM	A pre-existing cryptic donor within *BAP1* exon 4 (NM_004656.3) is strengthened, leading to a significant increase in its use. This mutation leads to a 27 nt deletion in the mutated exon 4 mRNA.

Example mutations which alter splicing in tumor-associated genes found in patients with these tumor types. Mutations are hyperlinked to their ValidSpliceMut Beacon page, which provides additional material such as IGV images of the RNAseq evidence for the regions of interest. GRCh37 coordinates are indicated

## Dataset validation and discussion

We have derived a GA4GH-standardized searchable resource for a large set of validated mRNA splicing mutations present in diverse tumor types. All variants passing QC in TCGA and ICGC cancer patients were analyzed with the Shannon pipeline ^19^. This revealed that 1,094,749 variants were predicted to have significant impacts on normal mRNA splicing (380,852 natural and 752,472 cryptic splice sites; 38,575 affecting both types). Subsequent RNA-Seq analysis with Veridical ^4^ provided evidence of abnormal gene expression specifically associated with a subset of these variant(s), identifying 341,486 unique mutations.

The molecular phenotype of each case containing a variant is classified in the ValidSpliceMut database and these results are collated for multiple tumors as the consensus phenotype. The molecular phenotype algorithm for Veridical-flagged natural splice site mutations finds 34.7% (87,382) of cases are classified as ‘aberrant’ mRNA isoforms, while 35.3% (88,993) and 30.0% (75,588) are deemed ‘likely aberrant’ and ‘allele-specific alternative splicing’, respectively. For cryptic site activating mutations, 28.7% (158,011) of cases were designated ‘aberrantly spliced’, 26.6% (146,520) as ‘likely aberrant’, and 44.7% (246,207) as ‘allele-specific alternative splicing’. Cases associated with natural site mutations are more likely to be classified as ‘aberrant’ when compared with cryptic splice site changes, due to the number of potential isoforms that could be generated by cryptic splicing mutations. Activation of cryptic sites or exon skipping is constrained by the relative strengths and locations of the cryptic versus their cognate natural sites ^6, 9^.

The vast majority of variant classifications of cases, when present in multiple tumors, are highly consistent across the same and distinct cancer types ( *Dataset 2*) ^28^. Nearly 75% of all mutations found in two or more tissues had a consistent molecular phenotype between >92% of samples with said mutation. The high proportion of samples with consistent molecular phenotypes across samples remains true when considering mutations that occur more frequently in ValidSpliceMut. For example, 77% of the variants present in >10 distinct tumor types have the same molecular phenotype across 94% of patients with said mutation. The consistency of molecular phenotypes of natural and cryptic splicing mutations suggest pleiotropy for this mutation category across multiple neoplastic tissues. Thus, the effects of splicing mutations on mRNA detected in one cancer diagnosis will likely be similar to those found in other tumour types.

Our results contrast with an earlier TCGA study that investigated alternative mRNA splicing ^29^ and demonstrated a limited set of non-constitutive exon-exon junctions attributable to cis-acting splicing mutations (n=32). The 3,311 novel or rare variants from TCGA patients that we report specifically activate abnormal cryptic splicing (significant ‘junction-spanning cryptic site use’ reads found by Veridical). This exceeds the number reported in cited study that analyzed all available TCGA tumor transcriptomes (n=1,914) ^30^. When ICGC datasets were included, a total of 3,650 variants were found to activate cryptic splicing. Comparing the validated cryptic splicing mutations we found with this previous report ^30^, 1,176 variants fulfilled our IT-based filtering criteria for constitutive splicing mutations. Veridical validated mRNA splicing effects for 824 of these variants (70.1%). The remaining 738 variants were reanalyzed for changes within the binding sites of regulatory splicing factors (SRF) that might affect normal mRNA processing. Together, including the effects on constitutive splicing, IT analysis of SRFs ^6^ (SRSF1, SRSF2, SRSF5, SRSF6, hnRNPA1, ELAVL1, PTB and TIA1) cumulatively identified changes in binding strength in 1746 (91.2%) sites affected by these variants.

Veridical validated splicing variants, which we define as mutations, were also tallied by tumor tissue type ( Table 1). 38.5% of unique mutations in TCGA (n=131,347) significantly weaken natural splice sites, while 65.0% (n=222,071) strengthen novel or pre-existing cryptic sites. 238,570 of these mutations (69.9%) are absent from dbSNP 150. 72,615 mutations (21.3%) are rare (found in <1% of the population), of which 28,812 (and those not present in dbSNP) were detected in multiple tumor types and cases. Valid mutations lacking rsIDs, by definition, represent either novel or recently observed variants. This low level of dbSNP saturation in TCGA is consistent with the possibility that many currently unknown mRNA splicing mutations may yet be discovered through additional sequencing studies.

**Figure 3.**
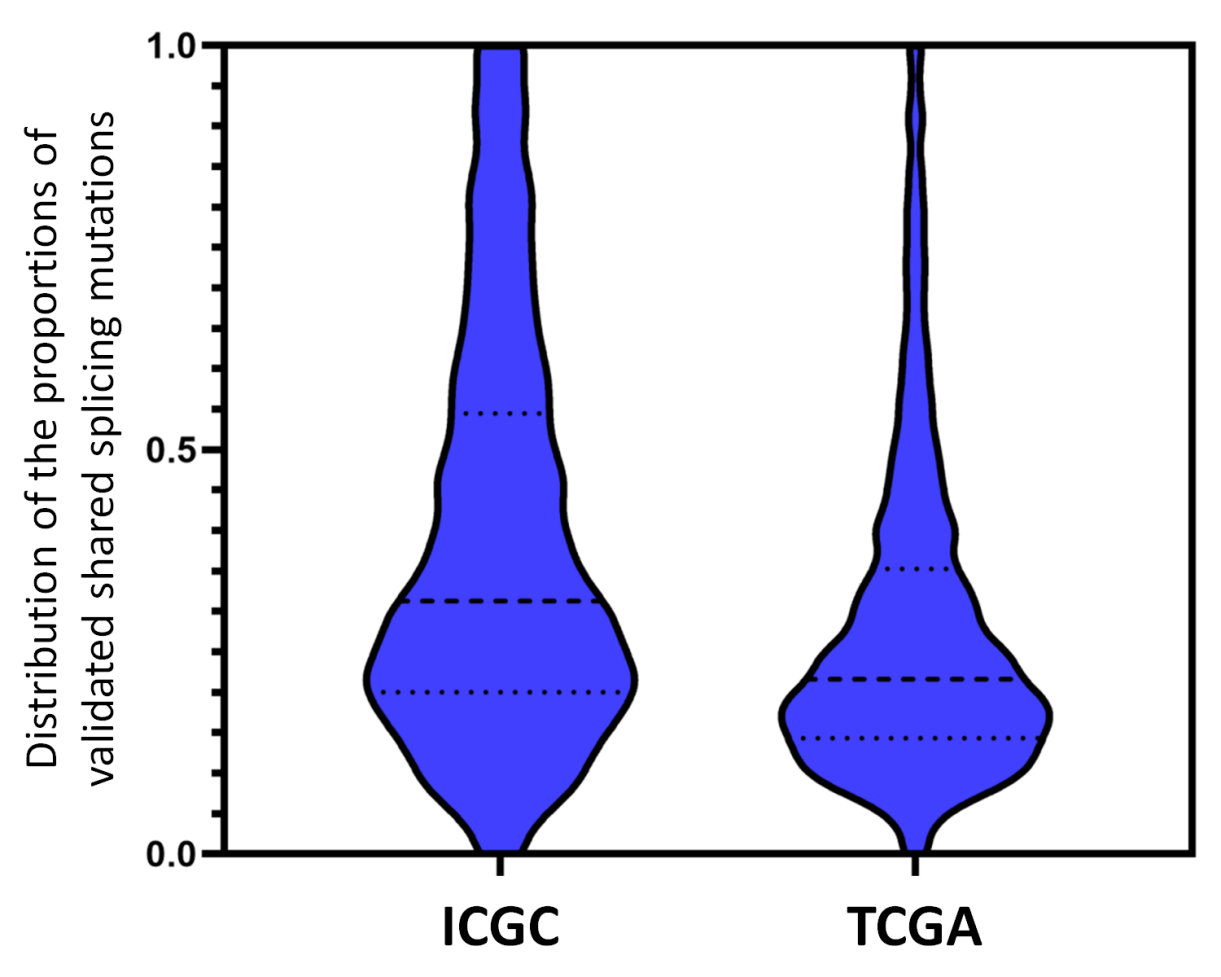
Census of Recurrent Splicing Mutations Present in Multiple ICGC and TCGA Patients. Predicted splicing mutations present in multiple tumors from the same dataset that cause splicing abnormalities were analyzed to determine validation rates, since such variants were less subject to technical artifacts, such as sequencing errors. Violin plots indicate the distributions of the fraction of predicted and validated splicing mutations present in multiple patients relative to the total number of tumours carrying those mutations in the TCGA and ICGA datasets. To achieve statistical significance (95% C.I.), distributions of 1,379 validated variants shared by both datasets and present in at least 9 ICGC (left) and 24 TCGA (right) patients were compared. A higher overall proportion of mutations are validated in the ICGC dataset (average of 38.6% for ICGC and 27.8% for TCGA). The dashed lines in each plot indicate the median (middle line), the upper and lower quartiles of the mutation fractions.

The ValidSpliceMut database consists of variants from both TCGA and ICGC, however the vast majority of variants were sourced from TCGA (329,758 of 341,597 flagged, unique variants were found only in TCGA patients; 96.5%). This had been expected, as the ICGC datasets were smaller (492 patients with available RNAseq data analyzed across 7 tumor types). There were 7,380 Veridical-flagged ICGC variants that were absent from TCGA patients; 4,459 variants were flagged in both TCGA and ICGC datasets (of which, 287 were not found in dbSNP 150). To evaluate the frequency of flagged TCGA and ICGC variants, we compared those shared between the two datasets that met SP criteria (n=9,485). We computed that for, a meaningful comparison (with 95% confidence interval [CI]), at least 9 ICGC and 24 TCGA patients should possess the shared mutation (typically, these correspond to common SNPs) and 1,379 shared variants met this criteria. We determined that on average, a higher average proportion of ICGC patients with shared mutations were flagged by Veridical compared to the TCGA cohort ( Figure 3). We observed that a higher fraction of SP-flagged variants are natural splice site alterations in the ICGC dataset compared to TCGA (49.7% to 38.3% of total SP-flagged variants, respectively), with fewer affecting cryptic sites (50.7% to 65.3%, respectively). A similar fraction of these sites were predicted to abolish natural splicing (16.8% ICGC and 14.4% TCGA of total SP-flagged variants). A higher percentage of ICGC variants compared to TCGA were confirmed with Veridical (49.1% to 30.9%, respectively), which may possibly be due to higher overall coverage in these regions in the RNAseq results for ICGC relative to TCGA. Interestingly, the fractions of novel variants i.e. not recorded in dbSNP, between TCGA and ICGC are inconsistent (70.0% vs. 42.4%, respectively). We speculate that this may be related to differences in sequence coverage, since TCGA variants were identified from a mixture of whole genome (WGS) and exomes ^16^, while ICGC variants were exclusively derived by WGS.

To assess the predictive accuracy of SP, we also analyzed an independent set of experimentally-validated splicing mutations that altered exon definition (1,050 variants), originally sourced from the Genome Aggregation Database; gnomAD ^31^ and validated with a high throughput, multiplexed splicing minigene reporter assay ^32^. Significant changes in constitutive splice site and/or (≥ 3 bit) SRF binding site strengths were found with SP for 1017 of these 1050 mutations (96.9%). Based on changes at constitutive splice sites alone, 447 variants were flagged (435 weaken natural sites, and 14 strengthen cryptic sites exceeding that of the most proximate natural splice site of the same polarity).

In Table 2, we highlight a representative subset of validated splicing mutations which were identified in known driver genes implicated in the COSMIC (Catalogue Of Somatic Mutations In Cancer) Cancer Gene Census catalog (CGC) ^33^. In total, 543 ‘Tier 1’ CGC genes exhibited at least one Veridical-flagged variant present in the ValidSpliceMut database. These mutations were associated with either increased exon skipping, intron inclusion, and/or cryptic site use. Mutations in Table 2 are hyperlinked to their corresponding ValidSpliceMut webpage, which provides additional information, including specific expression evidence for each of the tumors in the database that support predictions made by SP.

Many mutations generated multiple types of abnormal read evidence present in mis-spliced transcripts. Interestingly, a subset of TCGA mutations (n=33) produced evidence for every type of abnormal splicing reported by Veridical. *Dataset 3* ^34^ (see Data Availability) describes 11 representative mutations that simultaneously increase exon skipping, intron inclusion, and activate (or significantly increase use of) a strengthened cryptic site. In all but one instance, the mutation weakens the natural site, while simultaneously strengthening an adjacent cryptic site. The only exception involves the gene *SAP30BP*, where simultaneously occurring mutations in the same read (in linkage disequilibrium; separated by 4 nucleotides) independently cause two separate splicing changes: g.73702087G>A (c.661-1G>A; abolishes the natural acceptor of exon 10) and g.73702091G>A (c.664G>A; creates a weak cryptic acceptor site). The combined splicing impact of these variants is significant exon skipping, intron inclusion, and use of the activated cryptic site.

While ValidSpliceMut was designed to find evidence of variant-directed aberrant splicing, some variants with significant IT changes in splice sites are also associated with naturally-occurring, alternative splicing in controls. These are indicated by Veridical as non-wildtype splice junction reads in the index case that are not flagged as significantly different from controls (p-value ≥ 0.05). The relatively higher frequencies of these read in control samples is consistent with alternative mRNA splicing. The presence of the splicing mutation could theoretically alter the abundance of alternatively spliced isoforms. However, without evidence that disruption of the balance between constitutive and alternative forms would be deleterious, such variants are considered to exhibit a benign or likely benign molecular phenotype. The pre-filtered Veridical output was analyzed to identify variants that did not meet our p-value threshold to support validation of splicing mutation. We applied increasingly stringent thresholds to the average levels of alternative splicing in controls to identify alternatively spliced exons masquerading as predicted splicing mutations ( Table 3). A higher average number of reads in controls indicates that the alternative splicing events are more common in the control samples. Variants within the dinucleotides of intronic sequences at exon-intron boundaries have frequently been assumed to be pathogenic, and this analysis identifies those variants where this assumption may be incorrect. As expected, the most common alternative splicing events masquerading as splicing mutations were associated with exons that are commonly skipped or have reads which cross the exon-intron boundary ( Table 3). The ‘junction-spanning cryptic splice site use’ evidence type can only be computed as insignificant when the cryptic site is active in control samples. In all cases where this occurred (N=688), the cryptic site was functional in the wildtype sequence ( *R _i,initial_* > 1.6 bits [ *R _i,min_* ^7^]). These variants represent another type of alternately spliced mRNA via junction-spanning cryptic and exon skipping reads (average of ≥1 read per control), distinct from those in ValidSpliceMut, which have a similar classification, but does not explicitly determine read counts in control samples.

**Table 3.** Number of Unique Variants with Significant *R*_*i*_ Changes in Index Samples that also Exhibit Alternative Splicing in Controls*.

	Average Number of Reads per Set of Control Samples ^1^:			
Type of Junction-Spanning mRNA Read Evidence	≥5	≥3	≥1	>0
Cryptic Splice Site Use ^2^	313	392	563	688
Exon Skipping Reads ^2^	14,937	19,296	29,605	39,658
Intron Inclusion ^2^	232,276	303,256	433,881	507,609
Intron Inclusion with Variant Present ^2^	5,150	7,831	14,528	22,913

^1^ Standard deviation of control sample read average vary significantly between each variant. In some cases, a significant proportion of control samples will not have the read-type of interest despite a high average. ^2^ Includes variants that were not flagged by Veridical

This resource presents a set of splicing abnormalities in which we have the highest confidence because expression validation is required. We anticipate that some correct predictions of the Shannon pipeline may have not been validated by Veridical due to the limitations of mRNA detection; for example, either low expression of the gene harboring the mutation or nonsense-mediated decay of the corresponding transcript could be consistent with the effects of a valid splicing mutation, but in the absence of a sufficient number of abnormal reads, the mutation could not be confirmed. Indeed, expression of genes in tumours with validated splicing mutations exhibited greater overall gene expression than in other variants that were not validated in the same gene. The overall difference in gene expression between these two variant groups was statistically significant for 69.3% of genes based on the Student’s t-test (90% C.I.). Furthermore, overall gene expression in the group with Veridical-flagged variants exceeded the non-flagged group for nearly all other genes (99.7%). Differences between expression levels of Veridical-validated splicing mutations and other predicted mutations suggests the possibility that unverified SP predictions may arise from lack of or low levels of gene expression of the genes containing this subset of variants.

The original SP version that processed most of the TCGA data did not report regulatory splicing variants which influence exon definition. The Automated Splice Site and Exon Definition Analysis (ASSEDA) server ^6^ analyzes individual variants for regulatory and constitutive IT changes. Due to time constraints, it was not feasible to perform a reanalysis with the upgraded SP of the entire set of ~209 million unique variants present in the TCGA and ICGC datasets. However, the SP upgrade did verify improvements in detection of both constitutive and regulatory splicing mutations using the set of validated mutations reported in a recent study ^32^. This version of SP (available through our MutationForecaster system) is capable of predicting mutations in splicing regulatory sequences at higher throughput than ASSEDA and, like ASSEDA, accounts for relative abundance of mRNA isoforms by exon definition.

The Validated Splicing Mutation resource should significantly contribute to reducing the number of outstanding VUS in tumor (and possibly some germline) genomes, and substantially increases the number of functional variants with previously unappreciated consequences to mRNA splicing, in particular, those which activate cryptic splice sites. In our earlier study ^20^, a subset of the TCGA breast cancer patient data was evaluated with IT-based tools, identifying 988 mutations as significantly altering normal splicing by Veridical (19% of total mutations flagged by IT). This database greatly expands the size of the repository. Here, a higher ratio of rare or novel mutations have been validated by Veridical (31% of total mutations were flagged by IT). The higher yields seen here could be related to mutations present in multiple samples from the same tumor type and other tumor tissues, which would be expected to increase the probability of observing abnormally expressed splice forms for the same mutation.

## bioRxiv

An earlier version this article is available from bioRxiv: https://doi.org/10.1101/474452 ^35^

## Software availability

Archived code and scripts used as part of this study are available:

Zenodo: Validated Splicing Mutations Beacon API https://doi.org/10.5281/zenodo.1579898 ^22^

Zenodo: Validated Splicing Mutations Website https://doi.org/10.5281/zenodo.1579822 ^36^

Zenodo: Expression Data Processing, Histogram input generation and IGV Bash Script Generating Programs https://doi.org/10.5281/zenodo.1582421 ^37^

All software is licensed under a Creative Commons Attribution-Non Commercial-ShareAlike 4.0 International Public License

## Data availability

### Underlying data

*Zenodo*: **Dataset 1. Validated natural and cryptic mRNA splicing mutations.** Source data computed by the Shannon pipeline and Veridical, displayed on the ValidSpliceMut website ( https://validsplicemut.cytognomix.com/). DOI: https://doi.org/10.5281/zenodo.3377025 ^21^

*Zenodo:* **Dataset 2.** **Variant Distribution of Majority Molecular Phenotype Classifications Relative to All Classifications.** Supplementary figure containing six histograms (generated with GraphPad Prism v6.0). A variant may occur in multiple individuals, tissue types, or splice sites. Histograms present the fraction of molecular phenotype classifications which are consistent among all cases for variants found in at least 2, 3, 5, 10, 15, and 20 different tissue types. DOI: https://doi.org/10.5281/zenodo.3375706 ^28^

*Zenodo:* **Dataset 3. Mutations which lead to multiple types of aberrant splicing.** Representative set of mutations which significantly alter splicing in all evidence types analyzed by Veridical (i.e. cryptic splice site use, exon skipping, intron inclusion). Mutations are linked to their page on https://validsplicemut.cytognomix.com/, which provides additional material such as RNA-Seq images of the regions of interest. DOI: https://doi.org/10.5281/zenodo.1489941 ^34^

License: CC0 1.0

## Consent

Controlled-access TCGA and ICGC sequence data was approved by NCBI at the US National Institutes of Health (dbGaP Project #988: “Predicting common genetic variants that alter the splicing of human gene transcripts”; Approval Number #13930-11; PI: PK Rogan) and by the International Cancer Genome Consortium (ICGC Project #DACO-1056047; “Validation of mutations that alter gene expression”).

## References

[1] Foley SB Rios JJ Mgbemena VE: Use of Whole Genome Sequencing for Diagnosis and Discovery in the Cancer Genetics Clinic. *EBioMedicine.* 2015;2(1):74–81. 10.1016/j.ebiom.2014.12.003 26023681 PMC4444225

[2] Richards S Aziz N Bale S: Standards and guidelines for the interpretation of sequence variants: a joint consensus recommendation of the American College of Medical Genetics and Genomics and the Association for Molecular Pathology. *Genet Med.* 2015;17(5):405–424. 10.1038/gim.2015.30 25741868 PMC4544753

[3] Caminsky N Mucaki EJ Rogan PK: Interpretation of mRNA splicing mutations in genetic disease: review of the literature and guidelines for information-theoretical analysis [version 1; referees: 2 approved]. *F1000Res.* 2014;3:282. 10.12688/f1000research.5654.1 25717368 PMC4329672

[4] Viner C Dorman SN Shirley BC: Validation of predicted mRNA splicing mutations using high-throughput transcriptome data [version 2; referees: 4 approved]. *F1000Res.* 2014;3:8. 10.12688/f1000research.3-8.v2 24741438 PMC3983938

[5] Mucaki EJ Ainsworth P Rogan PK: Comprehensive prediction of mRNA splicing effects of BRCA1 and BRCA2 variants. *Hum Mutat.* 2011;32(7):735–742. 10.1002/humu.21513 21523855

[6] Mucaki EJ Shirley BC Rogan PK: Prediction of mutant mRNA splice isoforms by information theory-based exon definition. *Hum Mutat.* 2013;34(4):557–565. 10.1002/humu.22277 23348723

[7] Rogan PK Svojanovsky S Leeder JS: Information theory-based analysis of CYP2C19, CYP2D6 and CYP3A5 splicing mutations. *Pharmacogenetics.* 2003;13(4):207–218. 12668917 10.1097/00008571-200304000-00005

[8] Rogan PK Schneider TD: Using information content and base frequencies to distinguish mutations from genetic polymorphisms in splice junction recognition sites. *Hum Mutat.* 1995;6(1):74–76. 10.1002/humu.1380060114 7550236

[9] Rogan PK Faux BM Schneider TD: Information analysis of human splice site mutations. *Hum Mutat.* 1998;12(3):153–171. 10.1002/(SICI)1098-1004(1998)12:3<153::AID-HUMU3>3.0.CO;2-I 9711873

[10] Peterlongo P Catucci I Colombo M: *FANCM* c.5791C>T nonsense mutation (rs144567652) induces exon skipping, affects DNA repair activity and is a familial breast cancer risk factor. *Hum Mol Genet.* 2015;24(18):5345–5355. 10.1093/hmg/ddv251 26130695 PMC4550823

[11] Mucaki EJ Caminsky NG Perri AM: A unified analytic framework for prioritization of non-coding variants of uncertain significance in heritable breast and ovarian cancer. *BMC Med Genomics.* 2016;9:19. 10.1186/s12920-016-0178-5 27067391 PMC4828881

[12] Caminsky NG Mucaki EJ Perri AM: Prioritizing Variants in Complete Hereditary Breast and Ovarian Cancer Genes in Patients Lacking Known *BRCA* Mutations. *Hum Mutat.* 2016;37(7):640–652. 10.1002/humu.22972 26898890

[13] Yang XR Devi BCR Sung H: Prevalence and spectrum of germline rare variants in *BRCA1/2* and *PALB2* among breast cancer cases in Sarawak, Malaysia. *Breast Cancer Res Treat.* 2017;165(3):687–697. 10.1007/s10549-017-4356-8 28664506 PMC7032652

[14] Dos Santos ES Caputo SM Castera L: Assessment of the functional impact of germline *BRCA1/2* variants located in non-coding regions in families with breast and/or ovarian cancer predisposition. *Breast Cancer Res Treat.* 2018;168(2):311–325. 10.1007/s10549-017-4602-0 29236234

[15] Burke LJ Sevcik J Gambino G: *BRCA1* and *BRCA2* 5’ noncoding region variants identified in breast cancer patients alter promoter activity and protein binding. *Hum Mutat.* 2018;39(12):2025–2039. 10.1002/humu.23652 30204945 PMC6282814

[16] Hoadley KA Yau C Hinoue T: Cell-of-Origin Patterns Dominate the Molecular Classification of 10,000 Tumors from 33 Types of Cancer. *Cell.* 2018;173(2):291–304.e6. 10.1016/j.cell.2018.03.022 29625048 PMC5957518

[17] Global Alliance for Genomics and Health: GENOMICS. A federated ecosystem for sharing genomic, clinical data. *Science.* 2016;352(6291):1278–1280. 10.1126/science.aaf6162 27284183

[18] Fiume M Cupak M Keenan S: Federated discovery and sharing of genomic data using Beacons. *Nat Biotechnol.* 2019;37(3):220–224. 10.1038/s41587-019-0046-x 30833764 PMC6728157

[19] Shirley BC Mucaki EJ Whitehead T: Interpretation, stratification and evidence for sequence variants affecting mRNA splicing in complete human genome sequences. *Genomics Proteomics Bioinformatics.* 2013;11(2):77–85. 10.1016/j.gpb.2013.01.008 23499923 PMC4357664

[20] Dorman SN Viner C Rogan PK: Splicing mutation analysis reveals previously unrecognized pathways in lymph node-invasive breast cancer. *Sci Rep.* 2014;4:7063. 10.1038/srep07063 25394353 PMC4231324

[21] Mucaki EJ Shirley BC Rogan PK: Dataset 1. Validated natural and cryptic mRNA splicing mutations [Data set]. *Zenodo.* 2018 10.5281/zenodo.3377025 PMC6544075 31275557

[22] Shirley BC Mucaki EJ Rogan PK: Validated Splicing Mutations Beacon API (Version 1.0.0). *Zenodo.* 2018 10.5281/zenodo.1579898

[23] Su AI Wiltshire T Batalov S: A gene atlas of the mouse and human protein-encoding transcriptomes. *Proc Natl Acad Sci U S A.* 2004;101(16):6062–6067. 10.1073/pnas.0400782101 15075390 PMC395923

[24] Mucaki EJ Rogan PK: Expression changes confirm predicted single nucleotide variants affecting mRNA splicing. *bioRxiv.* 2019; 549089. 10.1101/549089

[25] von Kodolitsch Y Pyeritz RE Rogan PK: Splice-site mutations in atherosclerosis candidate genes: relating individual information to phenotype. *Circulation.* 1999;100(7):693–699. 10.1161/01.cir.100.7.693 10449689

[26] von Kodolitsch Y Berger J Rogan PK: Predicting severity of haemophilia A and B splicing mutations by information analysis. *Haemophilia.* 2006;12(3):258–262. 10.1111/j.1365-2516.2006.01216.x 16643211

[27] Vockley J Rogan PK Anderson BD: Exon skipping in IVD RNA processing in isovaleric acidemia caused by point mutations in the coding region of the *IVD* gene. *Am J Hum Genet.* 2000;66(2):356–367. 10.1086/302751 10677295 PMC1288088

[28] Shirley BC Mucaki EJ Rogan PK: Dataset 2. Variant Distribution of Majority Molecular Phenotype Classifications Relative to All Classifications. *Zenodo.* 2019 10.5281/zenodo.3375706

[29] Kahles A Lehmann KV Toussaint NC: Comprehensive Analysis of Alternative Splicing Across Tumors from 8,705 Patients. *Cancer Cell.* 2018;34(2):211–224.e6. 10.1016/j.ccell.2018.07.001 30078747 PMC9844097

[30] Jayasinghe RG Cao S Gao Q: Systematic Analysis of Splice-Site-Creating Mutations in Cancer. *Cell Rep.* 2018;23(1):270–281.e3. 10.1016/j.celrep.2018.03.052 29617666 PMC6055527

[31] Lek M Karczewski KJ Minikel EV: Analysis of protein-coding genetic variation in 60,706 humans. *Nature.* 2016;536(7616):285–91. 10.1038/nature19057 27535533 PMC5018207

[32] Cheung R Insigne KD Yao D: A Multiplexed Assay for Exon Recognition Reveals that an Unappreciated Fraction of Rare Genetic Variants Cause Large-Effect Splicing Disruptions. *Mol Cell.* 2019;73(1):183–194.e8. 10.1016/j.molcel.2018.10.037 30503770 PMC6599603

[33] Futreal PA Coin L Marshall M: A census of human cancer genes. *Nat Rev Cancer.* 2004;4(3):177–183. 10.1038/nrc1299 14993899 PMC2665285

[34] Mucaki EJ Shirley BC Rogan PK: Dataset 3. Mutations which lead to multiple types of aberrant splicing. *Zenodo.* 2018 10.5281/zenodo.1489941

[35] Shirley BC Mucaki EJ Rogan PK: Pan-Cancer Repository of Validated Natural and Cryptic mRNA Splicing Mutations. *bioRxiv.* 2018; 474452. 10.1101/474452 PMC6544075 31275557

[36] Shirley BC Mucaki EJ Rogan PK: Validated Splicing Mutations Website (Version 1.0.0). *Zenodo.* 2018 10.5281/zenodo.1579822 PMC6544075 31275557

[37] Mucaki EJ Shirley BC Rogan PK: Expression Data Processing, Histogram input generation and IGV Bash Script Generating Programs. *Zenodo.* 2018 10.5281/zenodo.1582421

