# Supplementary material for: Pan-cancer repository of validated natural and cryptic mRNA splicing mutations
Source: F1000Res. 2019 Sep 6;7:1908. Originally published 2018 Dec 7. [Version 3] doi: 10.12688/f1000research.17204.3 (PMC6544075; doi:10.12688/f1000research.17204.3)
Supplement: Peer review history

# Peer review: Reviewer response for version 3

**DOI:** 10.5256/f1000research.22439.r53578

**Published:** 2019-10-28

**Authors:** W.W.M. Pim Pijnappel

**Affiliations:** 1 Department of Pediatrics, Erasmus University Medical Center (Erasmus MC), Rotterdam, The Netherlands

I am happy with the changes, which have improved the manuscript. The only remaining comment is that I find the genome viewer image not so clear: in the example shown the exon skipping is difficult to see, it would help to show at least two or preferably 3 exons in the figure instead of a part of one exon, if possible.

I confirm that I have read this submission and believe that I have an appropriate level of expertise to confirm that it is of an acceptable scientific standard.

# Peer review: Reviewer response for version 2

**DOI:** 10.5256/f1000research.20308.r47751

**Published:** 2019-05-30

**Authors:** W.W.M. Pim Pijnappel

**Affiliations:** 1 Department of Pediatrics, Erasmus University Medical Center (Erasmus MC), Rotterdam, The Netherlands

The manuscript by the Rogan group potentially provides an interesting resource. I would like to suggest to make it more accessible for the user, because in its current form it is very difficult to judge how reliable and strong the splicing predictions and RNAseq data in reality are. Our lab has checked some variants that are know to affect splicing, and it is very difficult to judge how this resource rates these. For example, Ri before and after mutation are difficult to interpret, it is unclear from this manuscript what these values in practice mean. The text in figure 1 that describes the reads under individuals (bottom of A) does not always correspond to the IGV screenhot in B. It is also unclear what figure C actually shows. The term anti-cryptic has not been defined. It is also not clear to me to what extent the predictions were confirmed for the entire set and whether certain types of predictions were more accurate than others. The resource may be very valuable, I would recommend to make it more accessible and understandable for the biological and diagnostic researcher, who could greatly benefit from it.

I confirm that I have read this submission and believe that I have an appropriate level of expertise to confirm that it is of an acceptable scientific standard, however I have significant reservations, as outlined above.

# Response

**Published:** 2019-08-23

**Authors:** Peter Rogan

**Affiliations:** University of Western Ontario, Canada

Authors: We are grateful to the reviewer for these insightful comments, which we addressed below, in the manuscript and the ValidSpliceMut database.

Reviewer: The manuscript by the Rogan group potentially provides an interesting resource. I would like to suggest to make it more accessible for the user, because in its current form it is very difficult to judge how reliable and strong the splicing predictions and RNAseq data in reality are.

Response: To make this information more accessible to the user, we have made the following changes to the main text and to the ValidSpliceMut resource:

- Added a sentence which defines R *_i_* (see the next point)
- Added a “fold change” field to ValidSpliceMut, so users can understand how an information (R _i_) change is related to splice site binding strength (originally defined in reference #9).
- Added a Glossary of terms to ValidSpliceMut containing all information theory-based terms (R _i_, natural splicing) and Veridical terms (i.e. junction spanning, read abundance, etc) on the website, which were originally defined in the primary publication describing this algorithm and software (Viner et al., 2014).

The publication of the ACMG/AMP guidelines for interpretation of gene variants has accustomed users to simplified categorical characterizations, which has simplified clinical reporting. It should be recognized that a surprising number of variants in these databases have been reclassified recently, due to discordant interpretations and depositing of additional cases in ClinVar and other databases.

 We have developed an algorithm which classifies the molecular phenotypes of the variants reported in our database. Note that we do not attempt to perform clinical classification, since the variants we analyzed cannot be definitively causatively linked to the cancer phenotypes of the tumours from which they were derived. Our algorithm performs evidence-based case classification (view additional Methods and the Figure 2 flowchart for details), with thresholds/cutoffs calibrated to the phenotypes reported in ClinVar database shared by ValidSpliceMut. There are three categories: ‘aberrant’, ‘likely aberrant’, or ‘allele-specific alternative splicing’. This system is described in the manuscript in the ‘Methods’ section, and a categorical analysis of these classifications (i.e. proportion of natural site changes categorized as “aberrant”) are reported in the ‘Dataset validation and discussion’ section. We’ve updated Figure 1 to display the classification results using the same variant example in ValidSpliceMut that was shown in previous versions of this article.

 In this version of the manuscript, we also have added an additional analysis of the control population gene expression in which alternate splicing was evident at the site of the mutation (however, these exomes lacked the variant itself). This involved the analysis of variants that did not pass Veridical-based filtering steps (i.e. were not statistically significant). This analysis (presented in the ‘Dataset validation and discussion’ section) was performed completely independently of the previously described, splicing molecular phenotype classification algorithm.

Reviewer:  Our lab has checked some variants that are know to affect splicing, and it is very difficult to judge how this resource rates these. For example, Ri before and after mutation are difficult to interpret, it is unclear from this manuscript what these values in practice mean.

Response: We had not defined *R*_i_ here, as it had been described in our prior publications which are cited. However, for completeness, we have added the following statement in the main manuscript:

 “R *_i_* is a measure of binding site strength; it is related to affinity through the second law of thermodynamics and is measured in bits (a glossary of IT and Veridical terms can be found here). According to Shannon information theory, only sites with R *_i_* values exceeding zero bits can be bound. The minimum fold change in affinity is exponentially related to the difference in R *_i_* values of wild type and mutant binding sites (≥ 2 ^Δ *Ri*^). For example, a 3 bit change would result in at least an 8-fold change in binding affinity.”

 A glossary of terms to ValidSpliceMut has been included in the current version of the database to assist users in better understand the results of their queries. We also hyperlink to this glossary in the manuscript, where it is first mentioned.

Reviewer: The text in figure 1 that describes the reads under individuals (bottom of A) does not always correspond to the IGV screenshot in B.

               

Response: This discrepancy is due to a limitation on the number of reads which IGV can display at one time. This issue is most prevalent in regions of high coverage. While this is described in the main text, this explanation is given in the context of explaining why some IGV images may not display the splicing event at all.

 “In some cases, the splicing event observed by Veridical may not be present within the image window as the automated procedure used to create these images does not present all evidential sequence reads due to limitations on the number of reads that are shown.”

               

 This text has been edited to handle both instances where the IGV images is incomplete or absent:

 “In some cases, the splicing event observed by Veridical may not be present (or displayed in its entirety) within the image window as the automated procedure used to create these images does not present all evidential sequence reads due to limitations on the number of reads that are shown.”

 There are also cases where the user may observe more skipping reads than stated by Veridical. As mentioned in the manuscript, IGV images may include reads that appear to be exon skipping but are, in fact, activating cryptic splice site outside of the window range displayed. Although not explicitly mentioned, these false skipping reads could instead arise either from an alternate splice form that skips multiple exons or could be reads for a completely different gene overlapping the region. In these cases, Veridical will properly count only the reads skipping the exon of interest.

Reviewer  It is also unclear what figure C actually shows.

Response: The Tissue-specific Expression Histogram (Figure 1C) was included on ValidSpliceMut to allow the user to quickly determine the relative expression of variant-containing gene relative to the overall gene expression corresponding to the normal tissue from which the tumour was derived.  Expression levels are binned relative to all genes in the original dataset, and the bin containing the variant-containing gene is highlighted. This figure is relevant because the natural level of expressed at very low levels in a particular tumor type (or control tissue) may limit the number of reads observed, even if no mutation were present. A low basal level of expression impacts the confidence interval of the p-value reported by Veridical. The diagram in Figure 1C, for example, shows that *ATM* has a TPM (transcripts per kilobase millions) between 8-9 in mammary tissue (marked in purple; data from GTeX), and that this particular range of expression is low to moderate compared to the rest of the breast transcriptome.

 We have made slight adjustments to the Figure Legend:

 “C) A dynamically generated histogram presents expression levels of all genes for a selected normal tissue type. Genes are grouped into bins based on expression level, denoted on the x-axis. The number of genes present in each bin is shown on the y-axis (log _10_ scale). The histogram key indicates the ***expression range which contains ***the variant-containing gene ***(purple***). Tissue type can be changed via a drop-down list.”

Reviewer: The term anti-cryptic has not been defined.

Response: As this is a Data Note, we did not want to overexplain aspects of the Veridical software that had already been described elsewhere. As mentioned above, the manuscript cites the original Veridical paper ( Viner et al., 2014), which defines this term:

- Cryptic site use/anti cryptic site use: “Equivalent to: cryptic corroborating / non-cryptic corroborating reads”
- Cryptic corroborating reads: "Reads occurring within the expected region for cryptic splicing to occurring (i.e. spliced-in regions). This region is between the variant splice site location and the adjacent (direction dependent) splice junction."
- **Non-cryptic corroborating reads: "Also termed "anti-cryptic" reads. Not within the expected region, but still within the portion that would be expected to be spliced out, had cryptic splicing occurred."**

We also note that the Information icons (i) found next to each Veridical evidence-type in ValidSpliceMut direct the user to Viner et al. which define these terms. Finally, these terms are indicated in the aforementioned glossary on the ValidSpliceMut website.

Reviewer:  It is also not clear to me to what extent the predictions were confirmed for the entire set and whether certain types of predictions were more accurate than others.

Response: In general, lower p-values indicate a high likelihood that the frequency of aberrant splicing reads is caused by the variant. A lower p-value means higher confidence, as would a higher total number of aberrant splicing reads used to compute said p-value. While we provide p-values for all evidence types examined by Veridical, Viner et al. (2014) suggests that junction-spanning cryptic site use, exon skipping, intron inclusion (with mutation) and read-abundance intron inclusion are the most powerful evidence of variant-directed, aberrant splicing:

 “Not surprisingly, we have found that junction-spanning reads have the greatest value for corroborating cryptic splicing and exon skipping. Even a single such read is almost always sufficient to merit the validation of a variant, provided that sufficient control samples are used. For intron inclusion, both junction-spanning and read-abundance-based reads are useful, and a variant can readily be validated with either, provided that the variant-containing experimental sample(s) show a statistically significant increase in the presence of either form of intron inclusion corroborating reads.”

Reviewer: The resource may be very valuable, I would recommend to make it more accessible and understandable for the biological and diagnostic researcher, who could greatly benefit from it.

Response: Thank you. We hope the improvements that we have incorporated in the article and the website have made the resource easier to understand and use.

# Peer review: Reviewer response for version 2

**DOI:** 10.5256/f1000research.20308.r45974

**Published:** 2019-03-21

**Authors:** Emanuele Buratti

**Affiliations:** 1 International Centre for Genetic Engineering and Biotechnology (ICGEB), Trieste, Italy

I approve the amendments from Version 1.

I confirm that I have read this submission and believe that I have an appropriate level of expertise to confirm that it is of an acceptable scientific standard.

# Peer review: Reviewer response for version 1

**DOI:** 10.5256/f1000research.18813.r41665

**Published:** 2019-01-07

**Authors:** Francesca D. Ciccarelli

**Affiliations:** 1 Cancer Systems Biology Laboratory, Francis Crick Institute, London, UK; 2 School of Cancer and Pharmaceutical Sciences, King's College London, London, UK

The paper entitled "Pan-cancer repository of validated natural and cryptic mRNA splicing mutations" by Shirley, Mucaki  and Rogan describes an extensive analysis of pancancer somatic variants in samples of the TCGA dataset to identify mutations that affect splicing. To this aim, the authors combine the methods that they previously developed to predict mutations affecting splicing, with a validation of their effect on matched mRNA data from TCGA. 

 The study is technically sound and follows a line of investigation that has been a long standing interest of the authors. Despite this, I have a number of comments that hopefully will help strengthen the study:

1. The authors write that their IT-based framework to predict slicing variants "has been validated in multiple studies" and they refer to numerous papers. However, in all of them they act as co-authors, showing that their method is mostly used by themselves and their collaborators. This is not necessarily a problem, but it would certainly strengthen the study if the authors would perform a comparative assessment of their performance with other available methods to predict splicing mutations, for example those in dbNSFP. This will provide a less biased interpretation of the final results.
2. Somehow related to the previous point, the authors mention that their results "contrast with another TCGA study that investigated alternative mRNA splicing". In my opinion this point should be further explored: what are the main differences and what is the extent of overlap in concordant predictions? What are the possible reasons for these differences? This is important because the cited paper in Cancer Cell analysed the same dataset of mutations.
3. The authors notice that the number of variants which activate cryptic splicing exceed the number reported in a recently published study in Cell Reports. Similarly to before: what is the extent of overlap between the two datasets? Stating that a dataset is bigger than another one is not necessarily an indication that it is better.
4. The authors validate ~27% of predicted splicing variants using the mRNA data (351k validated of the 1.2M predicted). This is a surprisingly low fraction. Later in the manuscript, the authors briefly discuss about the possible reasons of such a discrepancy. One of them is the possible occurrence of nonsense mediate decay which will not confirm the mutations because no or very few reads will be detected. However, as the authors acknowledge, the absence of supporting reads only in mutated individuals as compared to the presence of reads in WT sample would be a strong indication of the effective role of these mutations on splicing. This can be quantified from the same RNAseq data and in my opinion should be done.
5. In general, the authors seem to exclude that their prediction method could lead to false positives. Rather they justify the poor overlap with limitations of mRNA detection. If this is the case, this should be quantified and probably a comparison with other prediction methods could help.
6. Of the >351k mutations with an effect on splicing supported by RNA data, only 35 affect CGC genes. Is this only a subset of mutations affecting driver genes or is it the complete list? In the former case, I would suggest that the authors provide the full list as supplementary data. In the latter case, the authors should discuss the implication of such a low number. Considering that there are >700 CGC genes, does it mean that aberrant slicing is very rarely a driver event? Is the overwhelming majority of splicing variants passenger?

I confirm that I have read this submission and believe that I have an appropriate level of expertise to confirm that it is of an acceptable scientific standard, however I have significant reservations, as outlined above.

# Response

**Published:** 2019-03-07

**Authors:** Peter Rogan

**Affiliations:** University of Western Ontario, Canada

We thank the reviewer for their valuable comments. Our responses follow:

1. The authors write that their IT-based framework to predict slicing variants "has been validated in multiple studies" and they refer to numerous papers. However, in all of them they act as co-authors, showing that their method is mostly used by themselves and their collaborators. This is not necessarily a problem, but it would certainly strengthen the study if the authors would perform a comparative assessment of their performance with other available methods to predict splicing mutations, for example those in dbNSFP. This will provide a less biased interpretation of the final results.

Response: We have previously compared our mutation prediction methods with others. Mucaki *et al.* Hum. Mut. 34:556-65 (2013) showed that IT-based single mutation and exon definition methods performed as well or better than MaxEntScan and Human Splice Finder websites. MaxEntScan computes relative entropy which is similar to IT, except it applies a correction for local base composition (which does not measure free energy, in contrast with IT: J. Theor. Biol. 201:87-92, 1989). Human Splice Finder does not measure changes in binding affinity and its basis is *ad hoc*. We also reviewed all articles (300+) which have used IT-based tools to predict changes in splicing (Caminsky *et al*., F1000Research 3:282, 2014). This reference covers the vast majority of studies that used IT-based bioinformatic tools for mutation analysis and, compared results obtained using these tools with other available software. The cited studies included a large proportion of mutations that we, ourselves, did not coauthor, or were analyzed by others, removing an obvious source of bias.

Regarding potential bias in our results, the IT-based position weight matrices (iPWMs) of splice recognition sites that we derived and use are based on a comprehensive set of splice sites spanning all known coding genes (see appendix of Rogan *et al.* Pharmacogenetics & Genomics 13(4):207-18, 2003). Other bioinformatic methods for splice site detection are based on many fewer splice sites for PWMs and are much more likely to be subject to bias based on how those sites were chosen. Also, the determination of information content in natural and mutated splice sites obeys the second law of thermodynamics (Schneider, J. Theor. Biol. 189:427-41, 1997); information contents have been formally proven to be related to binding affinities of splice site to splicesomes and splicing factors. MaxEntScan differs from IT because it applies a correction for local base composition, which is energetically and biochemically irrelevant to binding site affinity, and is therefore, biased.

As comparisons of IT with other methods have been covered previously, and the F1000Research Data Note article format is intended to specifically present results using the new resource we describe, in our opinion, reevaluation of other algorithms would not add anything of value to this manuscript.

2. Somehow related to the previous point, the authors mention that their results "contrast with another TCGA study that investigated alternative mRNA splicing". In my opinion this point should be further explored: what are the main differences and what is the extent of overlap in concordant predictions? What are the possible reasons for these differences? This is important because the cited paper in Cancer Cell analysed the same dataset of mutations.

Response: The paper being referred to in the reviewer’s comment is Kahles *et al.*, Cancer Cell., 34(2):211–224.e62018. This paper reports: “In a joint analysis of *cis* and *trans* associations with 50% prior on each type, we identified 32 *cis*- and seven *trans*-sQTL (Bonferroni corrected p < 0.05).” Information regarding these cis- sQTLs can be found here:

https://api.gdc.cancer.gov/data/3920a044-6874-4049-8010-55f0922243b7.

However, the document that provided does not indicate that these are actual splicing mutations. The document contains SNP coordinates, but not sequence changes. An assessment of known rsIDs at these coordinates only accounted for only 7 of the variants in our database. In fact, none of the substitutions (nor the rsIDs) in the article could be related back to changes in mRNA splice strength. It is not possible to comparison the results presented in this paper to those in Kahles *et al*. (2018).

3. The authors notice that the number of variants which activate cryptic splicing exceed the number reported in a recently published study in Cell Reports. Similarly to before: what is the extent of overlap between the two datasets? Stating that a dataset is bigger than another one is not necessarily an indication that it is better.

Response:  Jayasinghe *et al.* Cell Rep.;23(1):270-281.e3 (2018) identified 2056 variants in TCGA patients which activated cryptic splice sites, of which 1964 were confirmed by manual review of RNAseq data using the Integrated Genome Viewer (IGV). The data provided (Supplementary Table S1; “Passed Manual Review” tab) was sufficient to perform a comparison with our results. We scanned their manually reviewed variants using the Shannon Pipeline (SP). Despite reporting 1964 manually reviewed mutations, 50 mutations are shared between multiple patients (there are 1914 unique mutations total). 1510 of these mutations were found to alter at least one mRNA splice site (natural or cryptic), of which 1176 met our SP filtering criteria (either decreased natural site strength or cryptic splice sites strengthened by ≥2 bits and exceeding the strength of the nearest natural site of the same polarity).

We considered the possibility that splicing mutations in Jayasinghe *et al.* that were not flagged by SP could have instead altered the strength of splicing regulatory factor binding sites (SRFs). The high-throughput IT-based variant analysis tools needed to address this question were not available at the time the TCGA genomic data were processed. We recently introduced a new version of SP which is capable of analyzing variants impacting both constitutive splice sites and SRFs (including SRSF1, SRSF2, SRSF5, SRSF6, hnRNPA1, ELAVL1, PTB and TIA1). Upon analysis of the variants in Jayasinghe *et al.* for IT-derived changes in natural, cryptic and SRF binding site strengths, 1746 of the 1914 (91.2%) were found to be significant. It is conceivable that remaining unclassified variants may affect binding by splicing factors for which we have not yet derived iPWMs (i.e. SRSF7).

Of the 1176 variants meeting our filtering criteria, 824 were flagged by Veridical (70.1%). Interestingly, 27 Veridical-flagged mutations alter the same genomic coordinate in different tumours and another 64 affect the adjacent genomic coordinate within the same splice site in other individuals. We further investigated the remaining mutations (those evaluated, but not flagged) to better understand the discrepancy. In approximately 10% of cases, Veridical did not find any alternative validating cryptic splicing events in the region that contrasted with the read distribution in the set of control transcriptomes. For example, chr9:35389842G>C in TCGA-CN-5370 was expected to abolish the natural acceptor site of *UNC13B* exon 24, however the read counts for intron inclusion in the RNAseq data were too sparse to be deemed significant (p=0.33).

Jayasinghe *et al.* also found mutations in TCGA patients that were not evaluated in our study. This could occur for either because the RNASeq BAM file for a particular TCGA patient failed to download, or the “key” file that associates the BAM file to its TCGA name was incomplete in some tumors (i.e. TCGA-CG-4436; TCGA-STAD), due to both the BAM file name and header lacking this information. This would not impact the accuracy of the data that is present in our database or that we report, only the level of concordance between our results and those of Jayasinghe *et al*.

When comparing this dataset with our own, we discovered an instance where discrepant RNAseq data for the same tumour in the same TCGA patient led to different IT results. Originally, Veridical did not find a significant splicing change in *POLR1B* exon 14 at the +1 position, a G>A mutation g.113331138G>A, present in patient TCGA-Z6-AAPN in the ESCA dataset. When we manually reviewed the RNAseq BAM file used in this analysis, alternate or exon skipped splice forms were not observed, confirming the results reported from Veridical. In fact, TCGA deposited two separate BAM files containing RNAseq data for this same patient: “TCGA-Z6-AAPN-01A-11R-A406-31_rnaseq.bam” and “UNCID_2681450.b17c4505-8a84-4cdd-8782-fbc456deb2a6.sorted_genome_alignments.bam”. The latter BAM file shows evidence of both predicted exon skipping and activation of a cryptic acceptor site 12 nt downstream of exon 14 of *POLR1B*. Comparison of SNPs between these BAM files in other genes did not show any evidence of sample switching or contamination. Although this issue does not appear to be widespread in the TCGA dataset, such discrepancies exceed the scope of our study, and rightfully should be addressed by TCGA.

Nevertheless, this discovery did prompt us to re-analyze the TCGA-ESCA variants through Veridical using the second set of BAM files, and we have now included those results to ValidSpliceMut. In this new set, the previously mentioned *POLR1B* mutation is deemed significant due to increased exon skipping (86 reads showing exon skipping; p=0.0000).

4. The authors validate ~27% of predicted splicing variants using the mRNA data (351k validated of the 1.2M predicted). This is a surprisingly low fraction. Later in the manuscript, the authors briefly discuss about the possible reasons of such a discrepancy. One of them is the possible occurrence of nonsense mediate decay which will not confirm the mutations because no or very few reads will be detected. However, as the authors acknowledge, the absence of supporting reads only in mutated individuals as compared to the presence of reads in WT sample would be a strong indication of the effective role of these mutations on splicing. This can be quantified from the same RNAseq data and in my opinion should be done.

In the revision of this paper, the fraction of validated predicted splicing variants in the ValidSpliceMut database increased from 27% to 31%, as a result of a series of improvements in the software used for processing. SP was significantly upgraded over the course of this project,. The previous iteration would incorrectly report information changes at pre-existing splice sites adjacent to certain mutations. These sites were characterized by genomic coordinates that were altered by insertions or deletions (indel), regardless of whether the site overlapped or included the sequence change. In such instances, some altered natural splice sites could be designated as cryptic sites. SP also reported changes at cryptic acceptor and donor sites in the first and last exons of a gene, respectively, which were not likely to have a meaningful impact on splicing. Therefore, all datasets processed prior to this upgrade were reanalyzed for indel variants. The TCGA ESCA dataset was reprocessed with a second set of RNAseq BAM files (see above response to point 3), which increased the fraction of flagged mutations for that tumor type. Finally, we processed an additional 7 tumor datasets from ICGC and included the validated mutations in our primary beacon database. The statistics of mutations, their distributions and support have been updated in the present version of the manuscript.

From our perspective, the proportion of variants validated is not “a surprisingly low fraction". The results reported here are consistent with our previous published studies (Dorman *et al.*, Sci. Rep. 4: 7063, 2014). Aside from NMD, as demonstrated below, some mutations that significantly alter splice site strength may not have been flagged by Veridical as a consequence of low levels of expression of the gene in the tumor (or controls) itself. Furthermore, Veridical cannot make an accurate assessment of the region of interest in control samples if these lack sufficient read abundance levels to determine the probability (p-value) of observing expression in the mutation-containing vs control samples. Also, our analysis did not take into account other impacts of the variant on sequences that influence exon definition, such as binding to splicing regulatory factors or mRNA secondary structure. In our response to point 3, we described a discrepancy in BAM file sources, which could also lead to a lower fraction of confirmed variants. Finally, miscalled variants (despite the stringent quality control criteria applied to select variants) could contribute to the fraction of variants not supported by Veridical analysis. Such technical artifacts have been shown to be quite common in exome sequencing in areas of the genome characterized by low mappability (from Shi *et al.*, Cell Rep. 2018 Nov 6;25(6):1446-1457, 2018): “Examination of the genomic locations of mutations revealed that 41.1% of the artifactual somatic mutations occurred in regions of low mappability compared with only 6.4% for the validated somatic heterogeneous mutations.”

As indicated in our response to point 3, the previous version of this paper only evaluated variants for their impact on constitutional mRNA splice sites and cryptic sites, and excluded impacts of mutations at splicing regulatory factor binding sites (SRFs). The scope and time required to assess SRFs precluded the reanalysis of all datasets for such changes. However, to address the issue raised by the reviewer, we evaluated the degree to which ignoring SRFs would affect the overall discovery of splicing-related variants. The updated version of the Shannon Pipeline (SP) with this capability was used to examine 1050 mRNA splicing variants that have been demonstrated to affect exon recognition (Cheung *et al.*, Mol Cell. 2019 Jan 3;73(1):183-194.e8). These splicing variants were experimentally validated using a high throughput, multiplexed splicing minigene reporter assay in that study. SP reported a change in splicing and/or SRF binding strength for 1017 of these 1050 mutations (96.9%; where change in SRF strength was ≥ 3 bits). After accounting for SRF location (e.g. exonic TIA1 sites were eliminated, since these have not been proven to have splicing effects; see Table 1 of Caminsky *et al.*, F1000Res.;3:282, 2014, for a full description of each SRF), the number of flagged variants was reduced to 940 (89.5%). Based on changes in constitutive splice site strength alone, 447 variants were flagged (435 weaken natural sites, 14 strengthen cryptic sites to a level exceeding the nearest natural site). Therefore, 46.3% of the constitutive mutations at natural or cryptic splice sites were also flagged by SP. This suggests that the lower predictive accuracy of SP in our original submission was, in part, due to the limitations in its ability to detect pathogenic mutations in SRF binding sites.  

We addressed the reviewer’s suggestion to compare expression of the same gene in tumours with Veridical-validated mutations with other tumors with SP-predicted mutations in the same gene that were not experimentally validated.  To perform the analysis, we obtained pre-processed mRNA expression data from the same RNAseq sources of TCGA patients from cBioPortal ( www.cbioportal.com; provisional datasets were used, which contained largest number of patients for each tumor type). We extracted these gene expression values with a software program we wrote that determined transcripts per million (TPM) for each gene containing a SP-flagged variant.  Expression values for gene present multiple times (due to the presence of multiple splice isoforms) were averaged for the particular tissue from which they were derived.

We separated mRNA expression values for each gene in TCGA patients into the Veridical-flagged vs. non-validated SP-predicted mutation categories, and performed a Student’s t-test on the two groups. The expression values of 58.2% of genes were statistically distinct with 90% confidence; >2 patients per category per gene). With at least 10 patients per category, the number of statistically different genes increased to 69.3%. Among these genes, *patients with Veridical-flagged variants had higher overall gene expression in 99.7% of cases*. These inherent differences in expression suggest that the failure to validate predicted mutations may be related to little or no expression of these genes in tumour and/or control samples, rather than to accuracy of IT prediction methods. Non-sense mediated decay could be responsible for the decreased expression of these mutated genes in the tumour genomes that carry them, or the failure to validate could be related to low levels of expression of these genes in the particular tissues from which the tumours were derived. This analysis is now described in the revised manuscript (‘Dataset validation and discussion,’ para. 8).

Note: The gene expression data from cBioPortal had some limitations: 4196 genes containing variants flagged by SP are not present in the mRNA expression datasets, though the vast majority occurred in non-coding RNAs (i.e. 145 microRNAs, 194 LINC RNAs) or other uncharacterized RNAs (e.g. 324 ‘LOC’ RNAs). Furthermore, certain TCGA patients that we analyzed were not available in cBioPortal among the available expression datasets (2 TCGA-BRCA, 18 TCGA-COAD, 19 TCGA-GBM, 1 TCGA-HNSC, 1 TCGA-LUAD, 119 TCGA-OV, 4 TCGA-READ, 4 TCGA-STAD, 4 TCGA-THCA, and 7 TCGA-UCEC patients). Nevertheless, sufficient data were obtained for the analysis that we carried out.

Furthermore, this analysis has other caveats, especially regarding the accuracy of RNAseq quantification between samples, even upon normalization of the data. The vast majority of TCGA tumor samples do not have a matched normal counterpart, and many TCGA tumor datasets (e.g. TCGA-LAML) do not provide any normal control RNAseq data at all. While we could perform an analysis in which the tumor is compared to a set of normals for the same tissue, variation in expression between different individuals would obfuscate evidence of any apparent NMD caused by the mutation. A 50% reduction of expression (or less) may not be observable under these conditions.

5. In general, the authors seem to exclude that their prediction method could lead to false positives. Rather they justify the poor overlap with limitations of mRNA detection. If this is the case, this should be quantified and probably a comparison with other prediction methods could help.

Response: We have quantified RNA expression in tumors for mutations that were validated by Veridical vs those which were not (see response to point 4). Regarding false positives: An extensive comparison of Information Theory to other bioinformatic programs which evaluate variants for splicing impact (MaxEntScan and Human Splice Finder) has been performed (Mucaki *et al.* 2013; Caminsky et al. 2014; see response to point 1). False positives are extremely rare because strength of binding sites (in bits) is directly related to their binding affinities; we have demonstrated that unused cryptic splice sites in the vicinity of natural splice sites are significantly weaker. Based on our experience and published analyses of a genome-wide site of binding sites (Rogan *et al.* 2003), such decoy splice sites are nearly always at least 4 bits (2^4 or 16 fold) weaker than sites that are actually recognized by spliceosomes.

The predictive accuracy of the IT methodology for detecting expression-validated mutations was determined to be 87.9% (762 of 867 variants from 122 different publications; changes to SRFs were included in this variant dataset; Caminsky *et al.* 2014). This value is similar to the predictions made to those of Cheung *et al.* (2019), where we predicted splice site and/or SRFs changes to 89.5% mutations experimentally validated to cause exon definition events.

The performance of IT-based methods for predicting splicing mutations has been well established over the past two and a half decades. Re-evaluation of its accuracy is not necessary, and this issue is, at best, only tangentially relevant, to the purpose of presenting the resource described in a Data Note article.

6. Of the >351k mutations with an effect on splicing supported by RNA data, only 35 affect CGC genes. Is this only a subset of mutations affecting driver genes or is it the complete list? In the former case, I would suggest that the authors provide the full list as supplementary data. In the latter case, the authors should discuss the implication of such a low number. Considering that there are >700 CGC genes, does it mean that aberrant splicing is very rarely a driver event? Is the overwhelming majority of splicing variants passenger?

Response: There are 25 CGC genes indicated in Table 2, however these were never intended to be interpreted to be a complete list of CGC genes with Veridical-flagged mutations. The table has been renamed to indicate that these are a set of representative mutations. In the previous version of this paper, the number of variants (“n=25”) did not indicate the total number of CGC genes. This has been removed and replaced with the actual number of CGC splicing mutations that were validated:

     “In Table 2, we highlight a subset of validated splicing mutations which were identified in known driver genes implicated in the COSMIC (Catalogue Of Somatic Mutations In Cancer) Cancer Gene Census catalog (CGC) ^27^. In total, 543 “Tier 1” CGC genes have at least one Veridical-flagged variant present in the ValidSpliceMut database.”

# Peer review: Reviewer response for version 1

**DOI:** 10.5256/f1000research.18813.r41666

**Published:** 2018-12-12

**Authors:** Emanuele Buratti

**Affiliations:** 1 International Centre for Genetic Engineering and Biotechnology (ICGEB), Trieste, Italy

The increasing amount of sequencing data that is being generated in many biological systems has represented a real challenge to researchers in terms of trying to link individual changes to a particular biological process. The attempt, described in this work, to use IT approaches to evaluate the potential biological significance can significantly contribute to fill this gap. The laboratory of Peter Rogan has a long standing and internationally prominent role in addressing the possible consequences of sequence variants on the pre-mRNA splicing process especially with regards to its connection with human disease. The ValidSpliceMut developed in this work presents a user friendly interface that allows users to manually search for a variant (by gene name or genome coordinate range) and obtain information with regards to its possible effect on splicing. This will greatly help to better appreciate the functionality of Variants of Unknown Significance that are currently abundant genomic and transcriptomic Atlases.

I confirm that I have read this submission and believe that I have an appropriate level of expertise to confirm that it is of an acceptable scientific standard.
